# Extracellular histone H3 facilitates ferroptosis in sepsis through ROS/JNK pathway

**DOI:** 10.1002/iid3.754

**Published:** 2022-12-28

**Authors:** Zhijun Han, Zhizhou Yuan, Linfei Shu, Tao Li, Fan Yang, Lei Chen

**Affiliations:** ^1^ Department of Urology Surgery Zhuzhou Central Hospital Zhuzhou Hunan Province China; ^2^ The Second Affiliated Hospital of Hainan Medical University Haikou Hainan Province China

**Keywords:** c‐Jun N‐terminal kinase, estradiol, ferroptosis, histones H3, reactive oxygen species, sepsis

## Abstract

**Introduction:**

Previous evidence realized the critical role of histone in disease control. The anti‐inflammatory function of estradiol (E2) in sepsis has been documented. We here intended to unveil the role of extracellular histone H3 in sepsis regarding cell ferroptosis and the role of E2 in a such mechanism.

**Methods:**

Clinical sample, cecal ligation and puncture (CLP)‐induced animal models and lipopolysaccharides (LPS)‐induced cell models were prepared for testing relative expression of extracellular histone H3 and E2 as well as analyzing the role of extracellular histone H3 and E2 in sepsis concerning cell viability, reactive oxygen species (ROS), and ferroptosis.

**Results:**

Under sepsis, we found increased ferroptosis and extracellular histone H3 content, but reduced E2 concentration. Extracellular histone H3 facilitated ferroptosis of human umbilical vein endothelial cells (HUVECs) induced by LPS through activating the ROS/c‐Jun N‐terminal kinase (JNK) pathway. Moreover, E2 antagonized the effect of extracellular histone H3 on LPS‐induced HUVEC ferroptosis and sepsis injury in CLP‐induced animal models.

**Conclusion:**

We highlighted that extracellular histone H3 facilitated lipopolysaccharides‐induced HUVEC ferroptosis via activating ROS/JNK pathway, and such an effect could be antagonized by E2.

## Key points

- Sepsis increases ferroptosis and extracellular histone H3 content, but reduces estradiol (E2) concentration.
- Extracellular histone H3 induces ferroptosis of human umbilical vein endothelial cells (HUVECs) induced by lipopolysaccharides (LPS).
- E2 antagonizes the effect of extracellular histone H3 on LPS‐induced HUVEC ferroptosis.
- Extracellular histone H3 promotes the ferroptosis of HUVECs by activating JNK pathway.
- E2 antagonized the effect of extracellular histone H3 on sepsis injury in animal models.

## Graphical abstract

We highlighted that extracellular histone H3 facilitated lipopolysaccharides‐induced human umbilical vein endothelial cells ferroptosis via activating reactive oxygen species/JNK pathway. Such effect could be antagonized by E2.

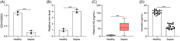

## 1 INTRODUCTION

Sepsis is a life‐threatening organ dysfunction as well as a heterogeneous syndrome is caused by the dysregulated host response to infection, and it acts as the contributor to mortality from infection.^1^ Each year, about 20 million people may suffer sepsis and over 5 million people may die of such disease with a mortality of about 26%.^1^ Evidence has pinpointed that the pathogenesis of sepsis is complex and shows a close correlation with various kinds of interaction between the infecting microorganisms and the host.^2^ Notably, the key point is noted that sepsis is an overreaction to infection and even the existence of severe infection or infection in the blood.^3^ Great improvements have been made in surgical and pharmacological modalities regarding sepsis therapy, but the increasing incidence of sepsis over the last 20 years has also been noted by epidemiological studies.^4^

Ferroptosis acts in varying roles in the known cell death pathways, pyroptosis, necroptosis, as well as apoptosis.^5^ Compelling evidence pinpoints the vital role of ferroptosis in the development and disease of various organisms.^6^ Specifically, ferroptosis exerts crucial functions in many pathological processes, including neurotoxicity, inflammation, heart disease, acute kidney failure, and liver injury.^7^, ^8^ Furthermore, the significance of ferroptosis in the development of sepsis has been documented.^9^

Histones are critical structural components of nuclear chromatin, and meanwhile, extracellular histones exert cytotoxicity and can result in immune damage.^10^ Additionally, extracellular histones produced upon inflammatory challenge may lead to endothelial dysfunction, organ failure, and even death during sepsis.^11^ Importantly, the crucial roles of extracellular histones in lung injury, brain, liver and kidney disease, peritonitis, thrombosis, sepsis, and autoimmune diseases.^12^ As earlier described, extracellular histones act as the main contributor to sepsis‐related death and specifically, the nuclear proteins histones H3 and H4 are cytotoxic.^13^

The vital role of estrogen in sepsis and its related injuries has been highly reported.^14^ Importantly, endogenous estrogen has been viewed as a causal or preventive factor in different diseases and cancers.^15^ Specifically, estradiol (E2) possesses anti‐inflammatory functions in sepsis.^16^ Under different stress conditions, the main function of E2 is to keep the oxidative phosphorylation system in mitochondria.^17^ The stressed mitochondria may result in excessive production of reactive oxygen species (ROS), which can cause damage in the mutate DNA and lipid bilayers, as well as changes in the activity of specific enzymes crucial for normal oxidative function.^18^ More importantly, induced ROS production and lipid peroxidation are the main features of ferroptosis.^19^

However, whether extracellular histone H3 and E2 involved in ferroptosis in sepsis remains to be explored. Here, we implemented animal and cellular experimentations for validation the significance of histone H3 and E2 in ferroptosis in sepsis.

## 2 METHODS

### 2.1 Clinical sample collection

Blood samples were collected from female patients with sepsis (*n* = 22) and healthy female volunteers (*n* = 22) enrolled in our hospital and stored at −80°C for further assay. The experiment was implemented in accordance with *Declaration of Helsinki* and ratified with Committee on the Ethics of Zhuzhou Central Hospital (No. 0301).

### 2.2 Animal model establishment

Male C57BL/6 mice specific pathogen‐free (20–25 g) were purchased from Hunan SJA Laboratory Animal CO., LTD. All animals were acclimatized for 1 week before experimentation and kept in temperature (22 ± 2°C) with a 12 h light/dark with free access to food and water. Sepsis was induced by cecal ligation and puncture (CLP), as previously described.^20^ Mice received sham operation were chose for the control. Modeled mice were treated with histone H3 (50 mg/kg), or estradiol benzoate (EB, 0.10 mg/kg). Tail vein blood was collected from each group of mice and prepared as serum samples for the detection of serum E2 and histone H3 levels. This study was undertaken according to the recommendations in the Guide for the Care and Use of Laboratory Animals of the National Institutes of Health and approved by the Committee on the Ethics of Zhuzhou Central Hospital (2018‐0358).

### 2.3 Hematoxylin and eosin (H&E) staining

After CLP operation for 48 h, the fresh tissues were collected and submitted to fixation utilizing 4% paraformaldehyde for 48 h, embedding in paraffin, sectioning and staining by H&E, followed by viewing with the help of a light microscope.

### 2.4 Enzyme‐linked immunosorbent assay (ELISA)

Human histone H3 and estradiol ELISA detection kits (USCN Life Science) were applied for determination of histone H3 and estradiol levels following the protocol of the supplier.

### 2.5 Measurement of reduced and oxidized glutathione (GSH/GSSG) ratio

GSH/GSSG ratio was determined in light of previously published method.^21^

### 2.6 Cell culture and treatment

Human umbilical vein endothelial cells (HUVECs) were purchased from American Type Culture Collection (CRL‐1730) and maintained in Dulbecco's modified Eagles Medium appended to 10% fetal bovine serum (Gibco), 100 U/ml of penicillin and streptomycin in a 5% CO_2_ incubator at 37°C. Cells were subjected to STR analysis before the experiments. The cells were subsequently diluted to 1 × 10^6^ cells/ml and seeded to six‐well plates followed by culturing for 48 h to 70% confluence and then reacted with 1 μg/ml lipopolysaccharides (LPS) or saline solution for 12 h before using. LPS‐exposed cells were further treated with histone H3 (Roche Life Science, Stockholm, Sweden) of different concentrations (20, 40, and 80 μg/ml), 17β‐estradiol (10 μg/ml, Sigma‐Aldrich) or SP600125 (10 μM, c‐Jun N‐terminal kinase [JNK] inhibitor, MCE).^22^

### 2.7 Cell counting kit8 (CCK8) assay

HUVECs (2 × 10^3^ cells/well) were seeded in 96‐well plates and incubated with the addition of 20 µl CCK‐8 solution (Jiancheng) at 37°C for 30 min. Then, the absorbance was tested at 490 nm followed by construction of related curves.

### 2.8 ROS content determination

Intracellular ROS production was measured with a ROS assay kit (Beyotime).

### 2.9 Measurement of iron content

An iron assay kit (cat. no. ab83366; Abcam) was employed for testing iron content according to the manufacturer's protocol.

### 2.10 Western blot analysis

Total proteins were prepared with the lysis buffer. Equal amounts of protein were separated by sodium dodecyl sulfate‐polyacrylamide gel electrophoresis and transferred onto a polyvinylidene fluoride membrane (Millipore). Membranes were submitted to blockage with 5% skim milk for 2 h at 37°C and incubation overnight at 4°C with the following primary antibodies: GPX4 (1:500, cat. no. PAS79321; Thermo Fisher Scientific, Inc.), ACSL4 (1:1000, ab155282; Abcam), p‐JNK (1:500, 4668, Cell Signaling Technology), JNK (1:500, 9258, Cell Signaling Technology), and GAPDH (1:1000, ab181602, Abcam). Membranes were then re‐probed with appropriate secondary antibodies for 1 h at ambient temperature, followed by development utilizing enhanced chemiluminescence (Millipore) and imaging on an Image Quant LAS 4000C gel imager (GE).

### 2.11 Statistical analysis

All results are expressed as means ± standard deviation, and were analyzed by one‐way analysis of variance or a student *t*‐test using SPSS 20.0 software (SPSS, Inc.). Values of *p* < .05 were concluded as statistically significant. Three repetitions were implemented at least.

## 3 RESULTS

### 3.1 Sepsis increases ferroptosis and extracellular histone H3 content, but reduces E2 concentration

**Figure 1:**
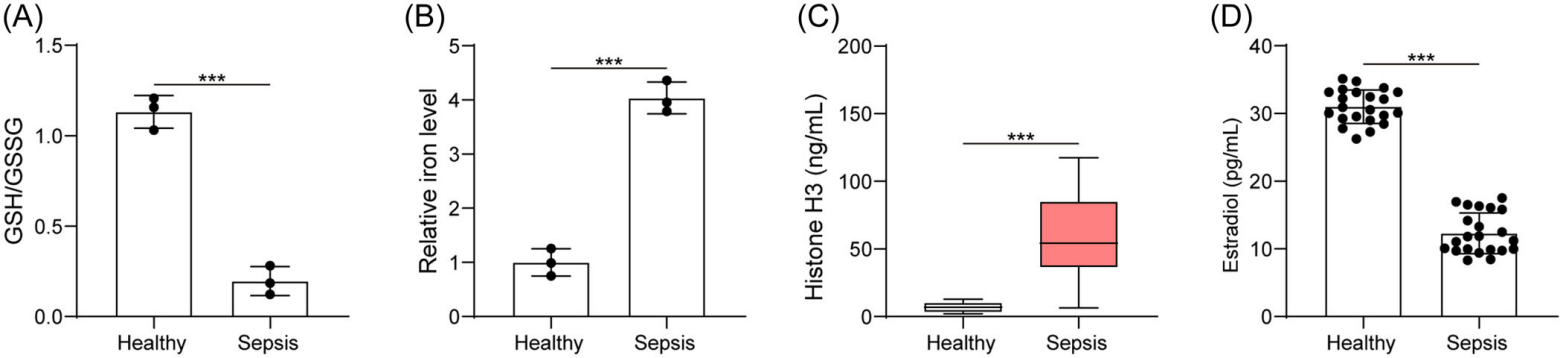
Sepsis increases ferroptosis and extracellular histone H3 content, but reduces E2 concentration. (A) Determination of GSH/GSSG in serum sample of healthy subjects and sepsis patients; (B) determination of iron content in serum sample of healthy subjects and sepsis patients; (C) determination of histone H3 content in serum sample of healthy subjects and sepsis patients by ELISA; (D) determination of E2 level in serum sample of healthy subjects and sepsis patients by ELISA. All results are expressed as means ± standard deviation, and were analyzed by a student *t*‐test. ***p < .001. *n* = 22. E2, estradiol; ELISA, enzyme‐linked immunosorbent assay; GSH/GSSG, reduced and oxidized glutathione.

Sepsis probably presents a suite of response by the immune system to injury.^23^ After clinical sample collection, we found that sepsis patients had reduced GSH/GSSG ratio and elevated iron level (Figure 1A,B). Through ELISA, an enhancement in histone H3 content but a reduction in E2 level were seen in sepsis patients (Figure 1C,D). Thus, we found increased ferroptosis and extracellular histone H3 content, but reduced E2 concentration in sepsis.

### 3.2 Extracellular histone H3 induces ferroptosis of HUVECs induced by LPS

**Figure 2:**
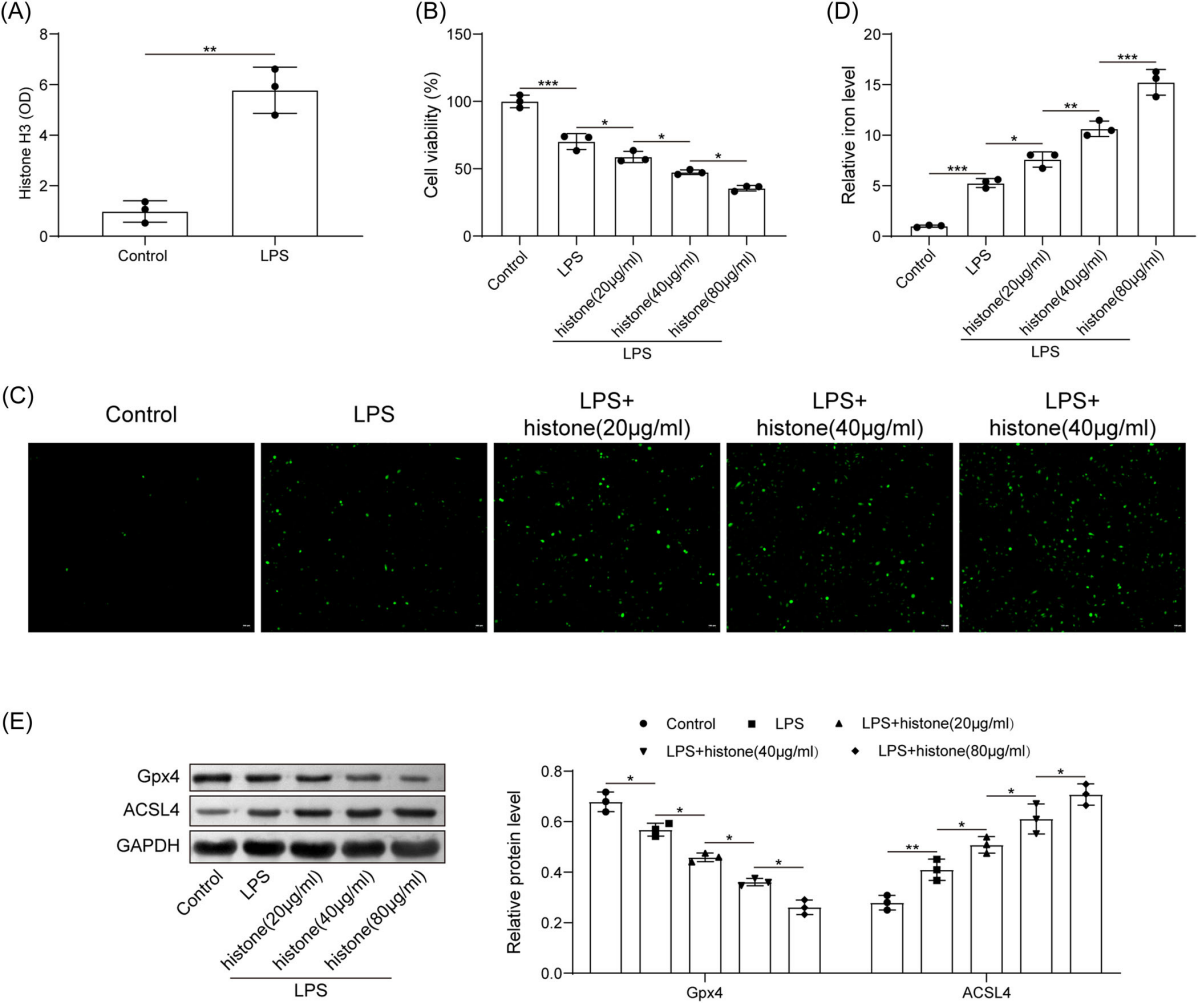
Extracellular histone H3 induces ferroptosis of HUVECs induced by LPS. (A) determination of histone H3 content in LPS‐exposed cells by ELISA; (B) detection of HUVEC viability after LPS or histone H3 treatment by CCK8; (C) determination of ROS level in HUVECs after LPS or histone H3 treatment; (D) determination of iron content in HUVECs after LPS or histone H3 treatment; (E) detection of Gpx4 and ACSL4 expression in HUVECs after LPS or histone H3 treatment by western blot analysis. All results are expressed as means ± standard deviation, and were analyzed by one‐way ANOVA. Three repetitions were implemented at least. *p < .05; **p < .01; ***p < .001. ELISA, enzyme‐linked immunosorbent assay; HUVECs, human umbilical vein endothelial cells; LPS, lipopolysaccharides.

Then, HUVECs were stimulated by LPS to mimic in vitro sepsis models. We identified from ELISA that LPS induction elevated histone H3 expression (Figure 2A). Through treatment of different concentrations of histone H3, we noted that LPS significantly inhibited cell viability, while histone H3 treatment further inhibited cell viability, and the higher the histone concentration, the lower the cell viability (Figure 2B). Moreover, LPS was able to induce ROS level and iron content, histone H3 treatment further elevated ROS level and iron content in a concentration dependent manner (Figure 2C,D). Further, LPS reduced the expression of Gpx4 and promoted the expression of ACSL4; with the increase of histone H3 concentration, the expression of Gpx4 was further downregulated, and the expression of ACSL4 was further upregulated (Figure 2E). Following experimentation, we selected 80 μg/ml for the subsequent assays. The aforesaid results concluded that extracellular histone H3 enhanced ferroptosis of HUVECs induced by LPS.

### 3.3 E2 antagonizes the effect of extracellular histone H3 on LPS‐induced HUVEC ferroptosis

**Figure 3:**
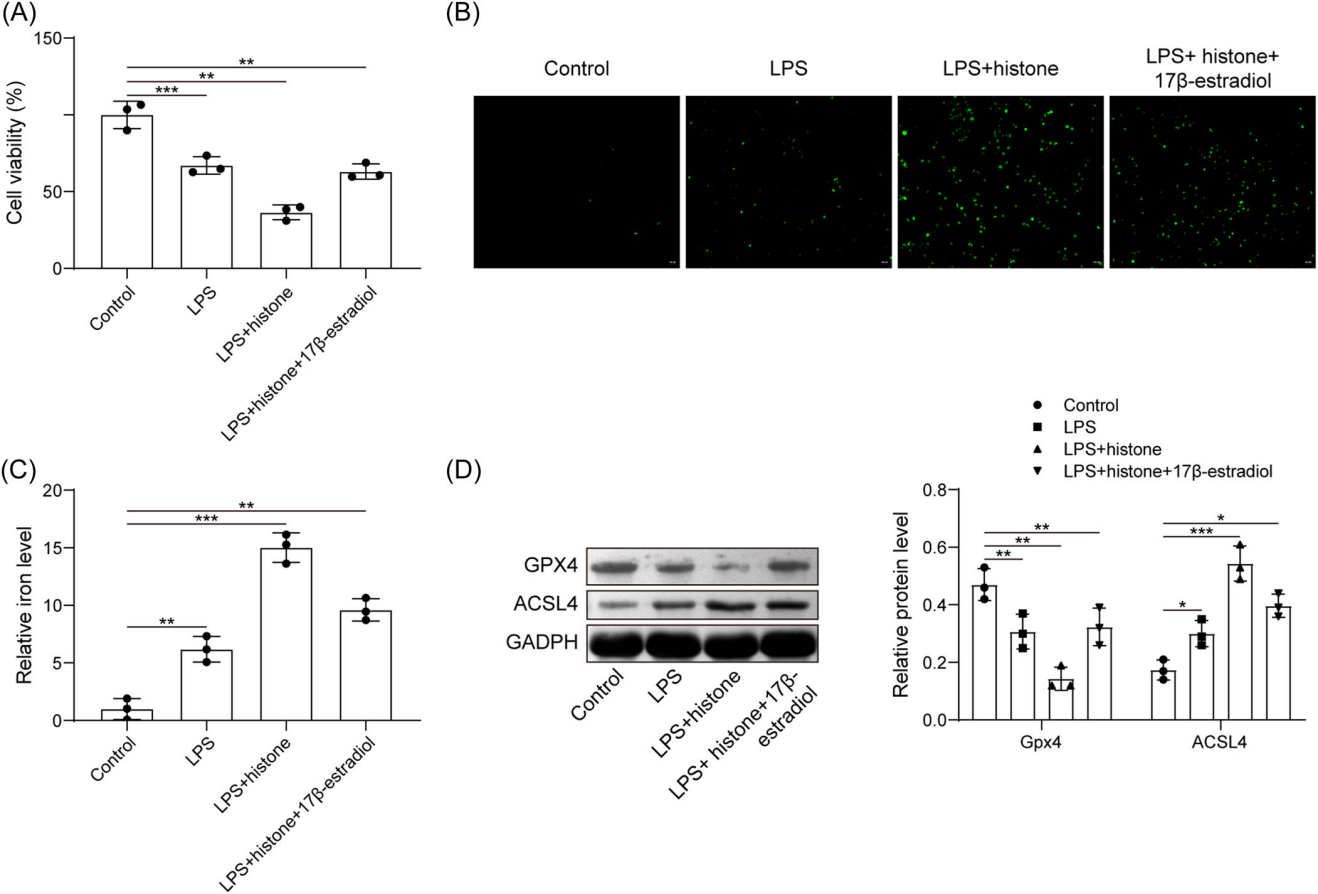
E2 antagonizes the effect of extracellular histone H3 on LPS‐induced HUVEC ferroptosis. (A) Detection of HUVEC viability after LPS, histone H3 or E2 treatment by CCK8; (B) determination of ROS level in HUVECs after LPS, histone H3 or E2 treatment; (C) determination of iron content in HUVECs after LPS, histone H3 or E2 treatment; (D) detection of Gpx4 and ACSL4 expression in HUVECs after LPS, histone H3 or E2 treatment by western blot analysis. All results are expressed as means ± standard deviation, and were analyzed by one‐way analysis of variance. Three repetitions were implemented at least. *p < .05; **p < .01; ***p < .001. E2, estradiol; HUVECs, human umbilical vein endothelial cells; LPS, lipopolysaccharides; ROS, reactive oxygen species.

Protective action of E2 in sepsis has been documented.^24^ Here, we focused on the role of E2 in LPS‐induced HUVEC ferroptosis. CCK8 assay clarified that compared with the LPS group, histone H3 further enhanced the inhibitory effect of LPS on cell viability, while E2 treatment significantly increased cell viability (Figure 3A). As shown in Figure 3B,C histone H3 further increased LPS‐induced ROS production and iron content, while further E2 treatment led to contrary trends. Further expression determination revealed that histone H3 inhibited the expression of Gpx4 and promoted the expression of ACSL4 in LPS‐exposed HUVECs; while further treatment with E2 brought about opposite trends (Figure 3D). Therefore, E2 was able to antagonize the effect of extracellular histone H3 on LPS‐induced HUVEC ferroptosis.

### 3.4 Extracellular histone H3 promotes ferroptosis of HUVECs by activating JNK pathway

**Figure 4:**
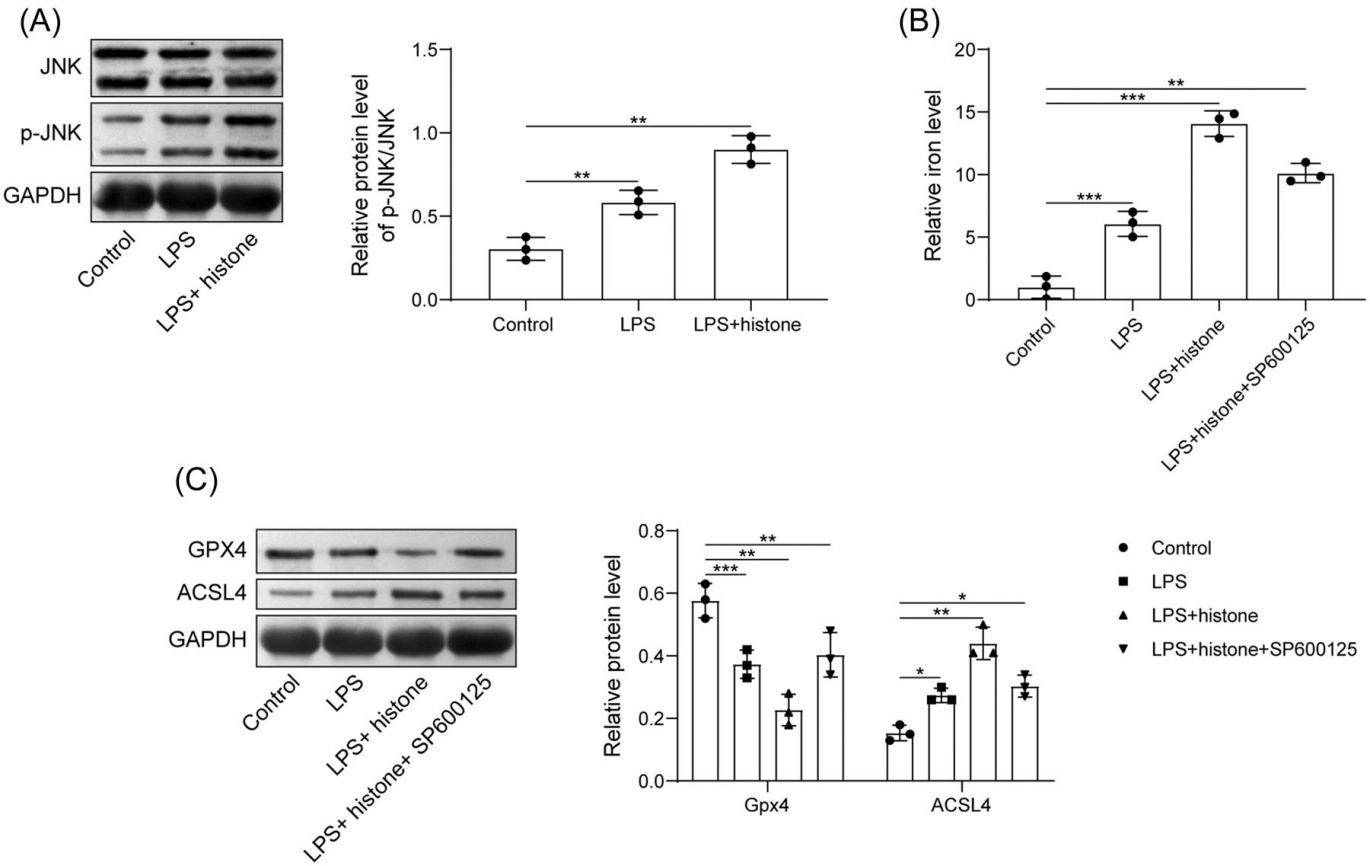
Extracellular histone H3 promotes the ferroptosis of HUVECs by activating JNK pathway. (A) Determination of p‐JNK/JNK expression after LPS or histone H3 treatment by western blot analysis; (B) determination of iron content in HUVECs after LPS, histone H3 or SP600125 treatment; (C) detection of Gpx4 and ACSL4 expression in HUVECs after LPS, histone H3 or SP600125 treatment by western blot analysis. All results are expressed as means ± standard deviation, and were analyzed by one‐way analysis of variance. Three repetitions were implemented at least. *p < .05; **p < .01; ***p < .001. HUVECs, human umbilical vein endothelial cells; LPS, lipopolysaccharides; ROS, reactive oxygen species.

We then tried to pinpoint the mechanism regarding the role of histone H3 in ferroptosis of HUVECs. We observed that LPS induction elevated expression of p‐JNK; while further histone H3 treatment further promoted the expression of p‐JNK. There was no significant change in the expression of JNK (Figure 4A). HUVECs were further treated with SP600125 (10 μM). Histone H3 increased the iron content and ACSL4 expression but reduced Gpx4 expression, while further addition of SP600125 caused opposite results (Figure 4B,C). Generally, extracellular histone H3 induced ferroptosis of HUVECs by activating JNK pathway.

### 3.5 E2 reverses the promoting effect of extracellular histone H3 on sepsis

**Figure 5:**
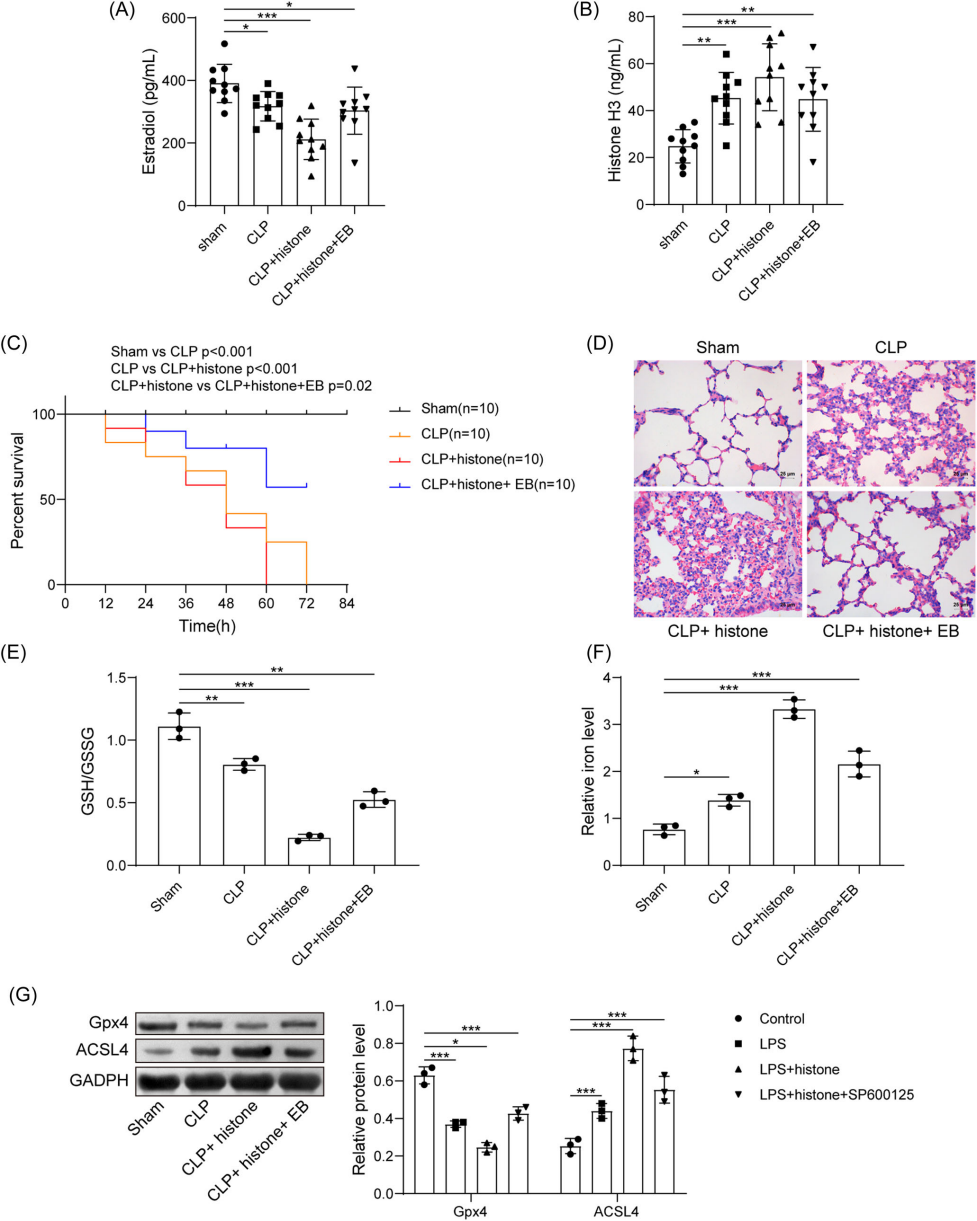
E2 reverses the promoting effect of extracellular histone H3 on sepsis. (A) Determination of serum E2 level in mice after CLP, histone, or EB treatment; (B) determination of histone H3 level in mice after CLP, histone, or EB treatment; (C) survival rate of mice after CLP, histone, or EB treatment; (D) pathological changes of lung tissues in mice after CLP, histone, or EB treatment; (E) determination of GSH/GSSG in lung tissues in mice after CLP, histone, or EB treatment; (F) determination of iron content in tissue homogenate in mice after CLP, histone, or EB treatment; (G) detection of Gpx4 and ACSL4 expression in lung tissues in mice after CLP, histone, or EB treatment by western blot analysis. All results are expressed as means ± standard deviation, and were analyzed by one‐way analysis of variance. *p < .05; **p < .01; ***p < .001. *n* = 10. CLP, cecal ligation and puncture; E2, estradiol; EB, estradiol benzoate;  GSH/GSSG, reduced and oxidized glutathione.

A sepsis mouse model was established for further validation. Determination of serum level of histone H3 and E2 depicted that CLP induction resulted in increased serum histone H3 and decreased serum E2 level. Histone H3 administration further increased serum histone H3 content and decreased serum E2 level. More importantly, EB administration could increase serum E2 level and decrease serum histone H3 level (Figure 5A,B). The survival rate of CLP‐induced mice was significantly reduced, which was further reduced by histone H3 treatment; while additional treatment with EB significantly prolonged the survival rate of modeled mice (Figure 5C). Observation from HE staining revealed that the morphology of alveoli in the sham‐operated mice was normal. CLP‐induced mice and CLP‐induced mice with histone H3 treatment showed vascular congestion and hemorrhage, the alveolar sac collapsed, the alveolar wall and the alveolar compartment were thickened. The lung tissue damage was more obvious in the CLP‐induced mice treated with histone H3. After further treatment with EB, the morphological changes of mouse lung tissue were significantly reduced (Figure 5D). Meanwhile, the contents of GSH/GSSG decreased in the lung tissue but iron contents increased in tissue homogenate of CLP‐induced mice, and the above changes were further enhanced by additional histone H3 treatment; while treatment with EB significantly reversed the above changes (Figure 5E,F). Further, a decline in Gpx4 expression but an increase in ACSL4 expression was seen in CLP‐induced mice and those treated with histone H3, while further EB treatment led to contrary findings (Figure 5G). Herein, E2 reversed the promoting effect of extracellular histone H3 on sepsis.

## 4 DISCUSSION

Sepsis is a systemic inflammatory response syndrome with high morbidity and mortality though early prevention and treatment of such disorder have been widely implemented.^23^ In the current study, we focused on the significance of histone H3 and E2 in ferroptosis in sepsis. Following in vitro and in vivo experimentations, we pinpointed that extracellular histone H3 promoted the ferroptosis of HUVECs by activating the JNK pathway, and moreover, such promotive function could be reversed by E2.

After clinical sample collection, we unfolded higher ferroptosis and extracellular histone H3 content, but reduced GSH/GSSG and E2 concentration in sepsis. In LPS‐induced in vitro sepsis model, subsequent to extracellular histone H3 treatment, LPS‐induced ferroptosis of HUVECs was enhanced, as evidenced by a decline in Gpx4 and an increase in ACSL4. Under sepsis, a lot of pro‐inflammatory and thrombogenic conditions affecting cells and organs can be attributed to extracellular histones which may gain from activated PMNs and macrophages.^25^, ^26^ Previous study also highlighted the cytotoxic action of histones H3 in sepsis as it can cause sepsis‐related death.^27^ GSH is confirmed as the first‐line defense aganinst oxidative stress, specifically, a reduction in GSH means the limited capability to eliminate free radical.^28^ Notably, the ratio of GSH/GSSG is a maker for evaluating the redox state of the cell or tissue.^29^ Oxidative stress is also a well‐studied inducer of ferroptosis, an iron‐dependent of nonapoptotic cell death.^30^ Moreover, Gpx4 is capable of limiting ferroptosis under normal conditions, and its downregulation during oxidative stress can induce cell death.^7^ Similarly, higher ACSL4 is also discovered by other clinical data in fatal cases of sepsis.^31^

Additionally, we uncovered that extracellular histone H3 promoted the ferroptosis of HUVECs by activating ROS/JNK pathway. Sepsis is known as an abnormal systemic inflammatory response modulated by the redundant generation of ROS and reactive nitrogen species.^32^ ROS are released by normal physiological processes and exerts crucial action in cell signaling and tissue homeostasis.^33^ Furthermore, ROS can activate JNK through many pathways.^34^ Notably, ROS is identified as a mediator for the inducer of the MAPK family members and possesses a vital effect on various biochemical roles and pathological conditions.^35^ As the major component of MAPK, extracellular JNKs can react to stimulation through modulation of cell ferroptosis, proliferation, apoptosis, and inflammation.^36^ More importantly, histone H3 is found to be modified at serine 10 and 28 in interphase cells under activation of the RAS‐MAPK or p38‐MAPK pathways with some growth factors or stress, and such modifications engage in the modulation of immediate‐early genes, whose elevation is a mark for multiple cancers.^37^

Further, E2 was concluded to be able to reverse the promoting effect of extracellular histone H3 on sepsis. Importantly, E2 is capable of modulating gene transcription through modifying epigenetic factors on histone proteins and DNA.^38^ The critical function of ROS as signaling molecules in multiple pathways modulating cell survival and death has been documented and accumulation of ROS is a feature of ferroptosis.^39^ Estrogen has been documented to limit the injury in the liver and intestines induced by sepsis in rats.^40^ Recent evidence has demonstrated estrogen limits sepsis‐induced liver injury by blocking ROS‐regulated NLRP3 activation.^41^ Moreover, E2 is capable of enhancing the trained immunity of women for fighting against sepsis.^42^ From the above‐mentioned evidence, we could believe the critical role of extracellular histone H3 in accelerating LPS‐induced HUVEC ferroptosis in relation to the ROS/JNK pathway. Moreover, E2 was able to reverse such an accelerating function. There is evidence that E2 is involved in the regulation of histone H3 acetylation and phosphorylation.^43^, ^44^ We speculate that E2 is also involved in the regulation of extracellular histone expression, thereby achieving its therapeutic effect in sepsis. Due to the limitations of the research conditions, we were unable to further explore the relationship between E2 and histone in this study, which may be studied in the future.

## 5 CONCLUSION

In conclusion, extracellular histone H3 could facilitate LPS‐induced HUVEC ferroptosis. Specifically, the induced role of extracellular histone H3 was achieved through ROS/JNK pathway, and moreover, such promotive action could be reversed by E2, pinpointing the significance of extracellular histone H3 and E2 in HUVEC ferroptosis in sepsis. However, the modulatory effect of extracellular histone H3 and E2 on the LPS‐induced HUVEC ferroptosis warrant further study in the setting of sepsis.

## AUTHOR CONTRIBUTIONS

Lei Chen was the guarantor of integrity of the entire study. Zhijun Han, Zhizhou Yuan, and Linfei Shu perform the study design did the experiment studies. Zhijun Han and Lei Chen wrote the manuscript. Tao Li did data analysis. Zhijun Han and Fan Yang prepared the figures. All authors reviewed the manuscript.

## CONFLICT OF INTEREST

The authors declare no conflict of interest.

## ETHICS STATEMENT

The collection of clinical samples is approved by the Ethics Committee of Zhuzhou Central Hospital (2018) ethics review (K) No. (0301). Animal experiments in this study is approved by the Committee on the Ethics of Zhuzhou Central Hospital (2018‐0358).

## Data Availability

The data sets used or analyzed during the current study are available from the corresponding author on reasonable request.
